# Bad Meat in Texas

**Published:** 1906-11

**Authors:** 


Bad Meat in Texas.—The October number of *Holland's Magazine* contains the results of analyses of thirty-one samples of meat purchased in Dallas, Fort Worth, Denison, Sherman, Houston, Corsicana, Corpus Christi, Cuero, Mexia, Yoakum, and Hillsboro.

In his article, "The Adulteration of Food in Texas," Walter B. Whitman says:

Five of these samples were of beefsteak and three of pork, none of which contained either preservatives or coloring matter. One of the steaks, however, had commenced to putrefy.

Three samples of sausages were examined; all contained boric acid and two of them also contained sulphites.

Two samples of Hamburger steak were examined and both found to contain sulphites.

Two samples of Bologna sausage contained boric acid, ferric oxide (coloring matter) and starch.

One sample of fish was free from both preservatives and coloring matter.

Fourteen samples of canned meats were analyzed and all but one showed the presence of either boric acid, starch or coloring matter; in some cases they contained all three.

Some of the canned meats were discolored by the action of chemicals on the cans, metallic compounds from the containers coating the meats. The can of potted ham purchased in Hillsboro was in a particularly bad condition, the meat being almost black.

The canned meats purchased included goods put up by different packers, mainly located in Chicago, but some of the samples were of goods canned in Texas. They were bought from grocers from their regular stock and the dealers were probably unaware of the fact that they were in any way preserved or adulterated.

